# Refractive and Biometric Outcomes in Patients with Retinopathy of Prematurity Treated with Intravitreal Injection of Ranibizumab as Compared with Bevacizumab: A Clinical Study of Correction at Three Years of Age

**DOI:** 10.1155/2018/4565216

**Published:** 2018-03-11

**Authors:** Yen-Chih Chen, San-Ni Chen, Benjamin Chi-Lan Yang, Kun-Hsien Lee, Chih-Chun Chuang, Chieh-Yin Cheng

**Affiliations:** ^1^Department of Ophthalmology, Changhua Christian Hospital, Changhua, Taiwan; ^2^School of Medicine, Chung Shan Medical University, Taichung, Taiwan; ^3^Department of Optometry, DaYeh University, Changhua, Taiwan; ^4^School of Medicine, National Taiwan University, Taipei, Taiwan

## Abstract

**Purpose:**

To compare refractive and biometric outcomes in patients with type 1 retinopathy of prematurity (ROP) treated with intravitreal injection of ranibizumab (IVR) versus bevacizumab (IVB), at a corrected age of 3 years.

**Methods:**

A retrospective case series compared cycloplegic refractive statuses and biometric statuses in patients who received either IVR or IVB for type 1 ROP, from April 2011 to April 2014.

**Results:**

A total of 62 eyes (33 patients) with type 1 ROP were evaluated (26 eyes in 13 IVR patients and 36 eyes in 20 IVB patients). There were no differences in birth statuses including gestational age and birth body weight between the two groups. The prevalence of refractive error greater than 1 D was higher in the IVB group (*p* = 0.03), and there was a higher prevalence of high myopia (<−5.0 D, *p* = 0.03) in the IVB group. Comparisons in biometric finding showed that IVB patients had shallower anterior chamber depth (*p* = 0.01).

**Conclusion:**

Both IVR and IVB showed low refractive errors, even followed at the corrected age of 3 years. No difference was noted between the two groups in refractive statuses. However, IVB was associated with shallower anterior chamber and higher prevalence of refractive error at the corrected age of 3 years. This trial is registered with NCT03334513.

## 1. Introduction

Retinopathy of prematurity (ROP) is a neovascular retinal disorder in preterm infants and is one of the major causes of childhood blindness. Although laser photocoagulation had been the main treatment for ROP, the Early Treatment for Retinopathy of Prematurity (ETROP) [1] study and other studies [2–4] have shown that laser photocoagulation results in high prevalence of both myopia and high myopia. Recent studies have reported the use of antivascular endothelial growth factor agents (anti-VEGF) to be effective in the treatment of type 1 ROP [5–7]. As for refractive outcomes, later studies have shown comparatively better refractive outcomes in bevacizumab-treated eyes versus laser-treated eyes [8, 9]. In addition to bevacizumab, intravitreal injection of ranibizumab (Lucentis; Genentech Inc.) has also been used for ROP treatment [10]. It has been proven that the use of ranibizumab can cause less systemic VEGF suppression as compared to bevacizumab [11]. Up to now, the efficacy of the 2 agents in regressing ROP has continued to be inconsistent. Some authors suggest that ranibizumab leads to a higher ROP relapse rate as compared to bevacizumab [12–14]. On the other hand, Chen et al. [15] have suggested that both agents have good efficacy without ROP regression in their cases. Interestingly, myopia was found to be more prevalent in the bevacizumab treatment group at 1 year follow-up. However, the results were only followed until the corrected age of 1 year. The long-term differences between the two medications in regard to the development of refractive errors have remained unknown. In addition, concerning refractive errors in ROP children, it is reported that the development of refractive errors is mainly influenced by anterior segment abnormalities such as steep corneal curvature, shallow anterior chamber depth or a relatively thick lens, rather than long axial length [16, 17]. But research into the differences in optic components between children receiving the two anti-VEGF agents has not yet been conducted. In this study, we aimed to investigate refractive outcomes over a longer follow-up at the corrected age of 3 years and compare effects on optical components of ROP patients between the two medications.

## 2. Materials and Methods

This is a retrospective study of type 1 ROP infants treated with IVI of anti-VEGF agents, in Changhua Christian Hospital, Changhua, Taiwan, from April 2011 to April 2014. The study was approved by the Institutional Review Board of the hospital and adhered to the Declaration of Helsinki. Only infants who received intravitreal injection of ranibizumab (IVR) or bevacizumab (IVB) as the primary treatment for ROP were included. Infants who received laser treatment whether as primary or as salvage treatment were excluded. Informed consent was obtained from the parents for the surgical procedures. The treatment indications were type 1 ROP disease, which was defined as zone I ROP with plus disease, zone I stage III ROP, and zone II stages 2-3 with plus disease according to the report of ETROP. The parents were well informed about the treatment choices, including laser treatment, or intravitreal injection of anti-VEGF agents. The efficacy and possible complications of each treatment were well explained. In addition, the difference between two anti-VEGF agents (ranibizumab or bevacizumab), such as different systemic VEGF suppression and differences in self-paid costs, were also well explained.

Medical records were collected in each treatment group for birth history data, including gestational age (GA), birth weight (BW), and postmenstrual age (PMA) at IVI. The zone and stage of ROP and corrected age at each visit were also recorded. All patients were evaluated routinely for refractive errors and optical components at the age of 1, 2, and 3 years. Cycloplegic refraction was performed at each visit, using a desktop computer auto kerato-refractometer (Topcon KR-8100, Tokyo, Japan), and was confirmed by retinoscopy examination. Refractive errors were calculated as spherical equivalent (SE) and astigmatism in cylinder. The average corneal radius (CR) was also measured (Topcon KR-8100, Tokyo, Japan). The biometric optic components, including anterior chamber depth (ACD), lens thickness (LT), and axial length (AL), were measured with an A-scan ultrasound (model A-1500; Sonomed, Lake Success, NY, USA). Refractive error was defined as follows: high myopia (SE ≤ −5 D), low myopia (SE > −5 D to −1 D), emmetropia (SE > −1 D to +1 D), low hyperopia (SE > +1 D to +4 D), and high hyperopia (SE > +4 D).

### 2.1. Surgical Technique and Follow-Up

The technique for IVI of anti-VEGF agents was as previous stated [13]. After topical anesthesia, the eyes were draped and disinfected with 5% povidone-iodine and topical antibiotic use. Either 0.625 mg (0.025 mL) of bevacizumab or 0.25 mg (0.025 mL) of ranibizumab was injected intravitreally 1.5 mm posterior to the limbus. After injection, intraocular pressure and retinal artery perfusion were checked and patients received topical antibiotics for 7 days. All patients were followed every 1 or 2 weeks following injection, until regression of ROP was observed. Then patients were scheduled for regular outpatient follow-up until at least a corrected age of 3 years. Indirect fundoscopy was performed to examine the retina and vascularization during each visit. The patients with poor response to anti-VEGF treatment or progression in ROP severity subsequently received laser treatment.

### 2.2. Statistical Analysis

Statistical analysis was performed using MedCalc software version 16.8.4 (MedCalc Software, Mariakerke, Belgium). Numerical data is expressed as mean ± standard deviation and median with 95% confidence interval. We used the Mann–Whitney *U* test to compare differences in baseline data (GA, BW, PAM at IVI, corrected age during follow-up), refractive status (SE, cylinder), and biometric components (AL, mean cornea radius, ACD, LT) between the two treatment groups. Fisher's exact test was conducted to compare differences regarding the ROP zones, stages, and presence of plus disease between the two treatment groups. It was also used to test the differences in the prevalence of refractive error between the two treatment groups. A *p* value < 0.05 was considered to be statistically significant.

## 3. Results

Table 1 listed the demographic data of our ROP patients. A total of 36 ROP patients were reviewed. Among these patients, 4 eyes from 3 patients in the IVB group had received photocoagulation treatment 2-3 weeks after the IVI of bevacizumab, due to failure of regression of retinal neovascularization, and were excluded from the study. Therefore, a total of 62 eyes in 33 patients (15 males and 18 females) were included in the study.

26 eyes from 13 patients belonged to the IVR group, and 36 eyes from 20 patients belonged to the IVB group. Among these patients, 9 of 62 eyes had zone I ROP, and 53 of 62 eyes had zone II ROP; 3 of 62 eyes had stage 2 ROP, and 59 of 62 eyes had stage 3 ROP. There were no differences in GA, BBW, or PMA at IVI, and corrected age at refraction and biometry was noted between the two groups (Table 2).

There were also no differences in the stage of disease or presence of plus disease or rubeosis iridis between the groups; however, eyes in the IVR group had a higher proportion of zone 1 disease (*p* = 0.02) (Table 3).

All studied eyes in both groups during subsequent follow-up showed complete regression of neovascularization, without recurrence. There were no major complications, such as traumatic cataract, retinal detachment, or endophthalmitis, found in the treated eyes.

**Figure 1:**
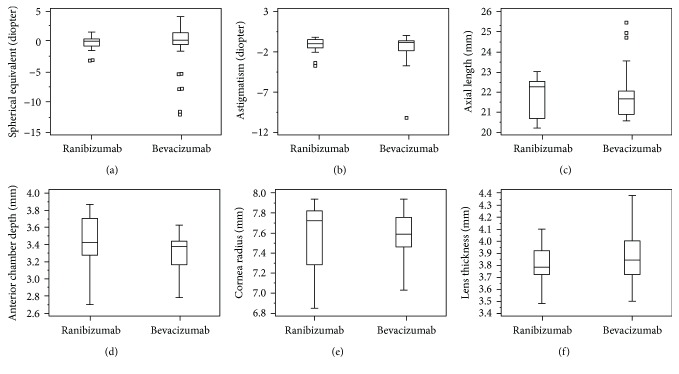
Box plots showing the distribution of refractive status and biometric comparisons at the corrected age of 3 years in children with ROP, treated with IVIs of either ranibizumab or bevacizumab. (a) Spherical equivalent; (b) astigmatism; (c) axial length; (d) anterior chamber depth; (e) cornea radius; (f) lens thickness. There was a significant difference only in anterior chamber depth (*p* = 0.01) between the eyes in the two groups but the differences were not seen in other parameters.

The distributions of SE, astigmatism, and biometric findings at the corrected age of 3 years in the ROP patients treated with either ranibizumab or bevacizumab are shown in Figure 1.  Table 4 gives a comparison in refractive errors and biometric findings between the two treatment groups.

The Mann–Whitney *U* test showed no significant differences in SE (−0.12 ± 1.12 D in the IVR group versus −0.65 ± 3.83 D in the IVB group, *p* = 0.19) or in cylinder power (−1.18 ± 0.89 D in the IVR group versus −1.60 ± 1.80 D in the IVB group, *p* = 0.55). Regarding the biometric studies, there was significantly shallower ACD in the IVB group (3.53 ± 0.22 mm in the IVR group versus 3.33 ± 0.23 in the IVB group, *p* = 0.01). There were no significant differences in cornea radius, LT, and AL between the two groups.

For the proportion of refractive errors in each group, patients in the IVB group had statistically significant higher proportion of high myopia and hyperopia (*p* = 0.03). Overall, IVB patients had a statistically higher proportion of refractive error greater than 1 D (*p* = 0.03) (Table 5).

## 4. Discussion

In this study, we followed the ROP patients for 3 years after the intravitreal injection of anti-VEGF agents. The results revealed that intravitreal injection of either bevacizumab or ranibizumab had good efficacy in the regression of ROP, up to a period of 3 years of age. Different from other previous studies, which showed that intravitreal injection of ranibizumab is related with higher incidence of ROP recurrence than bevacizumab [13, 14], our study showed that 4 eyes of 3 patients in the IVB group needed photocoagulation therapy for nonresponse, while no patients in the IVR group needed further therapy. In the comparison of refractive outcomes and biometric parameters in the two treatment groups, we found no differences regarding SE or astigmatism between the two groups at the corrected age of 3 years. However, a higher proportion of refractive error was found in patients of the IVB group as compared with patients in the IVR group. In our previous study [15], patients in the IVB group had a significantly higher incidence of high myopia (14.6% IVB versus 0.0% IVR) at the corrected age of 1 year. In this study, the higher incidence of high myopia was still noted at 3 years of corrected age (16.7% IVB versus 0.0% IVR). In addition, we noted a statically higher incidence of hyperopia in the IVB group and a higher incidence of refractive error greater than 1 D in the IVB group (*p* = 0.03 and 0.03, resp.). A higher chance of ametropia, including hyperopia, has also been reported in previous reports on ROP children [16, 17]. The possible mechanism may be related to the longer half-life of bevacizumab; this may increase the apoptosis of peripheral retinal structures, which are responsible for the normal emmetropization process [18]. This is because inhibition of VEGF receptors has been found to lead to a loss of Müller cells, astrocytes, and ganglion cells from the inner retina in an animal study [19]. However, the effect in human eyes remains unclear. Previous studies [20–22] have shown that anterior segment abnormalities (including a steeper cornea, shallower anterior chamber, and greater lens thickness, rather than axial length elongation) were the factors contributing to refractive errors in ROP children given laser treatment. In our study, we found that children in both groups had similar cornea curvature, lens thickness, and axial length. However, a shallower anterior chamber depth in the IVB group was found. This may partially explain the higher incidence of high myopia noted in the IVB group. Previous studies have shown that the arrested development of the anterior chamber may contribute to the development of myopia [23]. As compared to ranibizumab, the prolonged suppression of VEGF by bevacizumab may have a greater impact on the development of the anterior chamber; however, the mechanism needs to be further clarified in future studies.

## 5. Conclusion

In conclusion, we followed type 1 ROP patients who have received intravitreal injection of bevacizumab or ranibizumab as the main treatment, until a correction of 3 years. The results revealed good efficacy in ROP regression with both medications. The mean refractive error is similar between the two treatments, while patients receiving bevacizumab had shallower anterior chamber and higher incidence of ametropia. The difference in the duration of VEGF suppression may be responsible for the different incidence of ametropia and differences in anterior chamber depth between the two groups. The limitations of this study include the small sample size and the retrospective nature. Future studies with larger sample sizes and a prospective nature are needed to further confirm the results.

## Figures and Tables

**Table 1:** Demographic data of the patients.

Sex	Eye	Anti-VEGF	GA (weeks)	BW (g)	PMA (weeks)	Zone	Stage	Plus disease	Rubeosis iridis	Corrected age (months)	CYL (diopters)	SE (diopters)
F	OU	R	27	715	37	2/2	3/3	+/+	−/−	32	−1.750/−0.500	−0.125/0.000
M	OU	R	29	1402	40	3/3	3/3	+/+	−/−	36	−0.750/−1.250	0.125/−0.625
M	OU	R	25	832	34	2/2	3/3	−/−	−/−	35	−1.250/−0.500	−1.375/−1.250
M	OU	R	31	1350	36	2/2	3/3	+/+	−/−	30	−2.000/−1.250	−0.750/−0.625
M	OU	R	26	1138	35	22/	3/3	+/+	−/−	36	−0.750/−0.750	0.125/0.625
F	OU	R	26	521	39	2/2	3/3	+/+	−/−	37	−1.000/−0.750	1.000/0.375
M	OU	R	28	1115	39	2/2	3/3	+/+	−/−	31	−0.250/−1.000	0.125/0.250
F	OU	R	30	969	36	2/2	3/3	+/+	−/−	35	−0.500/−1.000	−0.500/−0.250
F	OU	R	25	758	35	2/2	3/3	+/+	−/−	38	−1.250/−2.000	0.875/0.250
F	OU	R	24	537	33	1/1	3/3	+/+	−/−	31	−0.750/−1.750	0.375/0.125
F	OU	R	26	507	33	1/1	3/3	+/+	+/+	39	−0.500/−1.500	0.500/1.000
F	OU	R	24	554	32	1/1	3/3	+/+	−/−	35	−3.750/−3.500	−3.125/−3.000
M	OU	R	24	732	40	1/2	3/3	+/+	−/−	37	−0.250/−0.250	1.625/1.125
M	OU	B	24	839	36	2/2	3/3	+/+	−/−	33	−0.750/−0.750	1.875/1.625
F	OD	B	26	780	33	2	3	+	—	37	−1.000	0.000
F	OU	B	25	720	36	2/2	3/3	+/+	−/−	34	−0.750/−0.500	2.625/2.250
M	OU	B	27	826	40	2/2	3/3	+/+	−/−	32	−1.500/−10.250	−0.250/−0.125
F	OU	B	24	686	35	2/2	3/3	+/+	−/−	37	−1.250/−0.750	−0.875/−7.875
M	OU	B	30	1247	35	2/2	3/3	+/+	−/−	35	−0.750/−0.500	0.375/−0.500
M	OS	B	28	974	46	2	3	+	—	34	−0.500	−0.250
M	OU	B	28	1039	38	2/2	3/3	+/+	−/−	35	−1.750/−1.500	−11.625/−7.750
M	OU	B	24	729	39	2/2	3/3	+/+	−/−	36	−2.000/−2.000	2.000/4.250
F	OU	B	26	724	35	2/2	3/3	+/+	+/+	36	−0.750/−0.750	2.125/2.375
F	OU	B	28	1060	36	2/2	3/2	+/+	−/−	38	−2.750/−1.500	−0.375/0.500
F	OD	B	27	954	42	2	3	+	—	37	−1.000	2.000
F	OU	B	25	751	35	2/2	2/2	+/+	−/−	34	−0.750/−0.500	0.125/0.250
F	OD	B	25	569	38	2	3	+	—	37	−1.750	0.375
F	OU	B	26	910	37	2/2	3/3	+/+	−/−	35	−2.500/−3.000	1.000/1.250
M	OU	B	28	1003	40	2/2	3/3	+/+	−/−	37	−0.500/−0.500	1.000/1.000
M	OU	B	32	1150	43	2/2	3/3	+/+	−/−	32	0.000/0.000	1.250/1.250
F	OU	B	32	1023	36	2/2	3/3	+/+	−/−	32	0.000/−1.250	0.750/0.375
F	OU	B	28	1120	32	2/2	3/3	+/+	−/−	39	−3.500/−3.250	−5.250/−12.125
M	OU	B	27	934	35	2/2	3/3	+/+	−/−	35	−3.750/−3.000	−5.375/−1.500

B: bevacizumab; BW: birth weight; CYL: cylinder; F: female; GA: gestational age; M: male; OD: right eye; OS: left eye; OU: both eyes; PMA: postmenstrual age; R: ranibizumab; SE: spherical equivalent.

**Table 2:** Comparison of demographic data between patients treated with ranibizumab or bevacizumab.

	Ranibizumab (*N* = 26)		Bevacizumab (*N* = 36)		*p* ^∗^
	Mean ± SD	Median (95% CI)	Mean ± SD	Median (95% CI)	
GA (weeks)	26.54 ± 2.28	26.00 (25.00 to 27.45)	27.06 ± 2.43	27.00 (26.00 to 28.0)	0.38
BW (g)	856.15 ± 306.46	758.00 (642.36 to 1034.87)	911.08 ± 175.17	922.00 (810.31 to 1009.82)	0.23
PMA at IVI (weeks)	36.12 ± 2.72	36.00 (34.55 to 38.45)	37.19 ± 3.28	36.00 (35.00 to 38.00)	0.26
Corrected age (months)	34.77 ± 2.83	35.00 (33.65 to 36.45)	35.08 ± 1.97	35.00 (34.00 to 36.00)	0.80

BW: birth weight; GA: gestational age; PMA: postmenstrual age; ^∗^Mann–Whitney *U* test.

**Table 3:** Disease status of ROP patients, by ranibizumab or bevacizumab treatment.

	Ranibizumab (*N* = 26)	Bevacizumab (*N* = 36)	*p* ^∗^
Zone, *N* (%)			0.02
I	7 (27%)	2 (6%)	
II	19 (73%)	34 (94%)	
Stage, *N* (%)			
2	0 (0%)	3 (8%)	0.25
3	26 (100%)	33 (92%)	

^∗^Fisher's exact test.

**Table 4:** Refractive errors and biometry in patients treated with ranibizumab or bevacizumab.

	Ranibizumab (*N* = 26)		Bevacizumab (*N* = 36)		
	Mean ± SD	Median (95% CI)	Mean ± SD	Median (95% CI)	*p* ^∗^
SE (diopters)	−0.12 ± 1.12	+0.13 (−0.36 to +0.38)	−0.65 ± 3.83	+0.38 (−0.17 to +1.09)	0.19
Cylinder (diopters)	−1.18 ± 0.89	−1.00 (−1.30 to −0.75)	−1.60 ± 1.80	−1.00 (−1.59 to −0.75)	0.55
Axial length (mm)	21.90 ± 0.94	22.33 (21.39 to 22.56)	21.90 ± 1.27	21.67 (21.31 to 21.95)	0.22
Cornea radius (mm)	7.64 ± 0.26	7.76 (7.45 to 7.83)	7.60 ± 0.22	7.60 (7.52 to 7.68)	0.35
ACD (mm)	3.53 ± 0.22	3.51 (3.35 to 3.74)	3.33 ± 0.23	3.37 (3.22 to 3.44)	0.01
Lens thickness (mm)	3.82 ± 0.15	3.78 (3.74 to 3.98)	3.85 ± 0.20	3.82 (3.78 to 3.94)	0.60

ACD: anterior chamber depth; SE: spherical equivalent; ^∗^Mann–Whitney *U* test.

**Table 5:** Presence of refractive errors of the ROP patients treated with ranibizumab or bevacizumab at the corrected age of 3 years.

	Ranibizumab (*N* = 26)	Bevacizumab (*N* = 36)	*p* ^∗^
High myopia (≤−5 D)	0 (0%)	6 (16.7%)	0.03
Myopia (high + low) (≤−1 D)	4 (15.4%)	7 (19.4%)	0.75
High hyperopia (>+4 D)	0 (0%)	1 (2.8%)	1.00
Hyperopia (high + low) (>+1 D)	2 (7.7%)	12 (33.3%)	0.03
Presence of ametropia (>+1 D or <−1 D)	6 (23.1%)	19 (52.8%)	0.03

^∗^Fisher's exact test.
